# A hybrid finite-discrete element method for modelling cracking processes in sandy mudstone containing a single edge-flaw under cyclic dynamic loading

**DOI:** 10.1038/s41598-024-66397-z

**Published:** 2024-07-03

**Authors:** Xiaolong Zhang, Wenjie Xu, Xiaoping Zhang, Yan Yu, Chong Xu

**Affiliations:** 1 National Institute of Natural Hazards, Ministry of Emergency Management of China (NINH, MEMC), Beijing, 100085 China; 2 State Key Laboratory of Hydroscience and Engineering, Tsinghua University, Beijing, 100084 China; 3 The Key Laboratory of Safety for Geotechnical and Structural Engineering of Hubei Province, School of Civil Engineering, Wuhan University, Wuhan, 430072 China; 4 Key Laboratory of Compound and Chained Natural Hazards Dynamics, Ministry of Emergency Management of China, Beijing, 100085 China; 5 Key Laboratory of Landslide Risk Early-Warning and Control, Ministry of Emergency Management of China, Chengdu, 610059 China

**Keywords:** Cohesive zone model, Hybrid FDEM, Quasi-static loading, Cyclic dynamic loading, Rock failure mechanism, Natural hazards, Civil engineering

## Abstract

Rock mass deformation and failure are macroscopic manifestations of crack initiation, propagation, and coalescence. However, simulating the transition of rocks from continuous to discontinuous media under cyclic dynamic loading remains challenging. This study proposes a hybrid finite-discrete element method (HFDEM) to model crack propagation, incorporating a frequency-dependent cohesive-zone model. The mechanical properties of standard sandy mudstone under quasi-static and cyclic dynamic loading were simulated using HFDEM, and the method's reliability was verified through experimental comparison. The comparative analysis demonstrates that HFDEM successfully captures crack interaction mechanisms and accurately simulates the overall failure behavior of specimens. Additionally, the effects of pre-existing flaw inclination angle and dynamic loading frequency on rock failure mechanisms were investigated. The numerical results reveal that rock samples exhibit significantly higher compressive strength under dynamic loading compared to quasi-static loading, with compressive strength increasing with higher cyclic dynamic load frequencies. Furthermore, by analyzing the strength characteristics, crack propagation, and failure modes of the samples, insights into the failure mechanisms of rocks under different frequency loads were obtained. This study provides valuable insights into crack development and failure of rocks under seismic loads, offering guidance for engineering practices.

## Introduction

**Figure 1:**
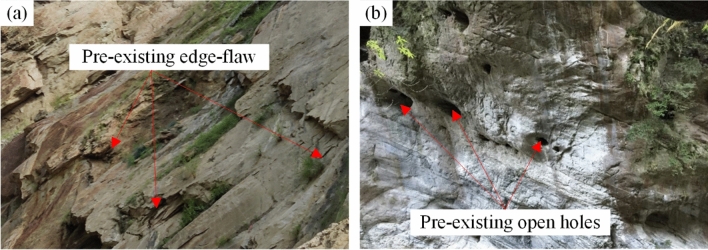
Examples of various flaws on rock slopes observed in the field. (**a**) Pre-existing edge-flaw (modified after Ref.^5^), (**b**) pre-existing open holes contained in rock masses (modified after Ref.^6^).

**Figure 2:**
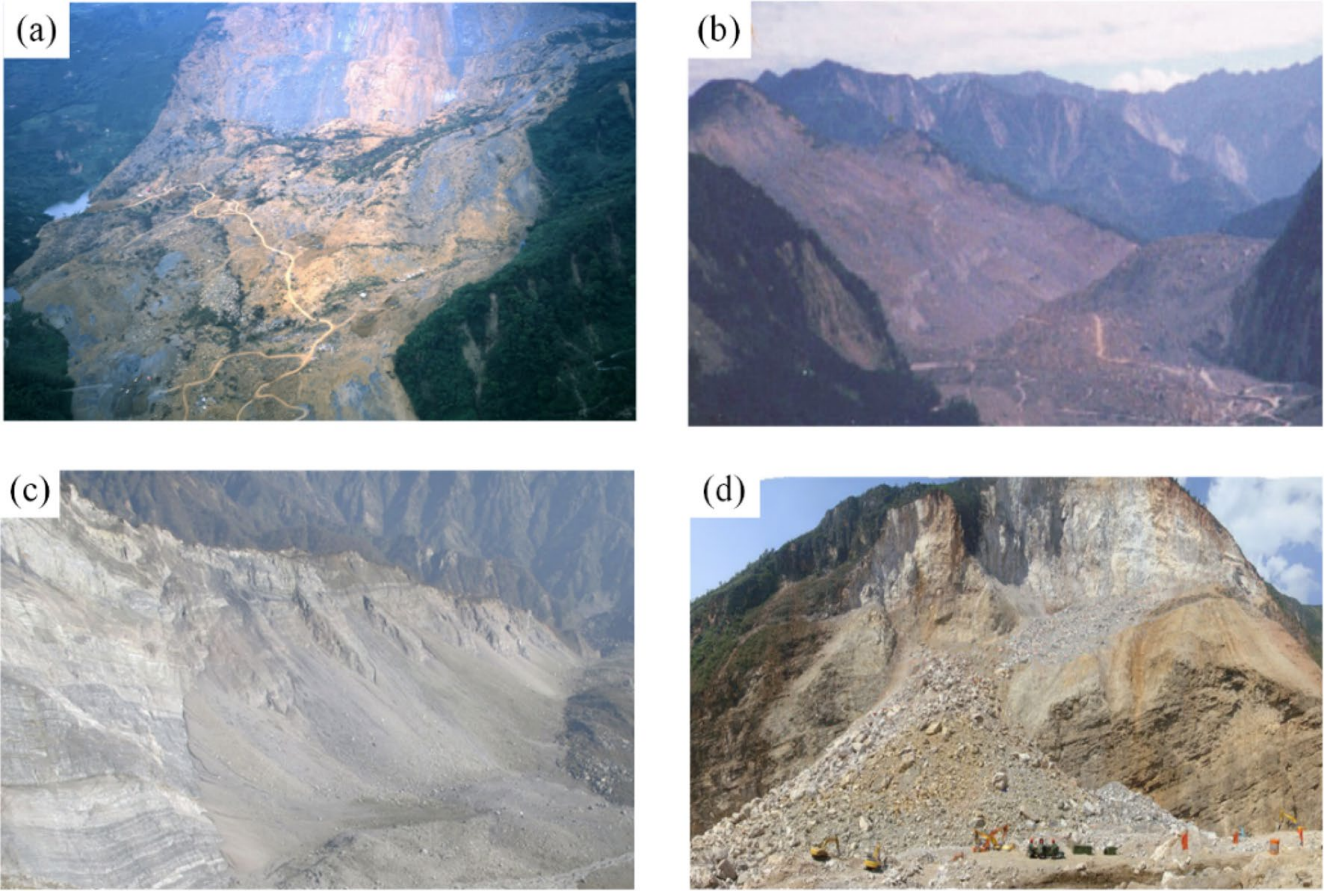
Landslides induced by earthquakes. (**a**) Chiu-fen-erh-shan landslide (modified after Ref.^1^), (**b**) Tsaoling landslide (modified after Ref.^2^), (**c**) Daguangbao landslide (modified after Ref.^3^), (**d**) Hongshiyan landslide (modified after Ref.^4^).

Rock is a special geological material that often exhibits flaws at varying scales on its surfaces and within its interiors due to prolonged geological processes (Fig. 1). These flaws can exist at different scales, range from microscopic cracks to larger fractures and discontinuities. It is evident that natural rock is a non-homogeneous material, and the cracks within it significantly affects its mechanical behavior. Among the potential hazards associated with rock formations, earthquake-induced slope failure is a prevalent natural geological disaster (Fig. 2). The failure of rock slopes is characterized by suddenness, strong destructiveness, high-speed movement, and long run-out, which often causes serious casualties and economic losses^1–4^. Hence, the rock fracture under the action of earthquakes is widely considered as one of the most important and hazardous engineering problems.

Earthquake-induced rock landslides usually occur in shallow layers of the surface. Hence compared to confining pressure, the internal joints and flaws in rock masses have a greater impact on the deformation, strength, and crack propagation form of the rock^7^. Then the stability of rock slopes is mainly controlled by internal joints and flaws in the rock mass, and the fracture process of the rock mass can be seen as a macroscopic manifestation of crack initiation and growth^8–12^. The fundamental reason for the rock slope failure under earthquake is the deformation and fracture of the rock mass under external load, which is essentially the process of crack initiation and growth under seismic load. However, due to the strong nonlinear characteristics of the deformation or fracture process of jointed rock masses, it is very difficult to predict the strength and failure mode of rock masses^13^. Therefore, a better understanding of the crack initiation and growth in rock mass is helpful to the study of the mechanical mechanism of rock slope failure under seismic loading^11^.

Up to data, many scholars have conducted uniaxial compression experiments on rock/rocklike specimens containing a single pre-exiting flaw^8,14–17^, double pre-exiting flaws^10,18–22^ or multiple pre-exiting flaws^23–30^ with different geometric shapes by means of laboratory experiments and numerical simulations to study the cracks initiation, propagation, and coalescence resulting in macroscopic failure of the specimen. In addition, the crack coalescence types, initiation angle, propagation modes, and fracture characteristics were further explored. However, due to the different materials and sizes of the specimens, the experimental results are varied. It should be noted that numerical simulation cannot completely replace laboratory testing at this stage or even for a long time in the future. But effective numerical methods are often beneficial supplements to experiments. The study of the crack initiation and growth rules of rock materials with pre-existing flaws under dynamic loading has important theoretical significance and engineering value for revealing the macroscopic nonlinear mechanical behavior of rock fracture process under earthquake action and explaining the engineering fracture phenomenon caused by crack propagation. However, there is relatively few research on the true fracture process of rocks containing pre-existing flaws under seismic loads, and resulting in an unclear understanding of it^11^. Even if some scholars have used some numerical simulation methods to study rock fracture modes at present, most of the research is focused on the situation under quasi-static loads^32–35^. The numerical simulation of rock transition from continuous to discontinuous media under dynamic loads remains a challenge.

Understanding the dynamic characteristics and failure mechanism of fractured rock masses under cyclic dynamic loading is crucial, therefore it is necessary to develop reliable numerical methods for accurate simulation. To address these challenges, this study presents a hybrid finite-discrete element method (HFDEM). The paper is organized as follows. "Fundamental theory of hybrid finite-discrete element simulation method" introduces the basic theory of the hybrid finite element discrete element simulation method, which introduces a frequency dependent cohesive zone model. The proposed method was validated in "Testing and validation of hybrid finite-discrete element simulation method" by comparing and analyzing the results of static and dynamic uniaxial compression tests. "Models for uniaxial compression of specimens with prefabricated cracked flaw" discusses the effects of the inclination angle of pre-existing edge-flaw and input seismic parameters on the damage mechanism of rock masses. Finally, the key conclusions of this study are drawn in "Conclusions". The proposed method is expected to provide valuable insights into the behavior of rocks under dynamic loading conditions, driving advancements in rock mechanics, geotechnical engineering, and seismic hazard assessment practices.

## Fundamental theory of hybrid finite-discrete element simulation method

**Figure 3:**
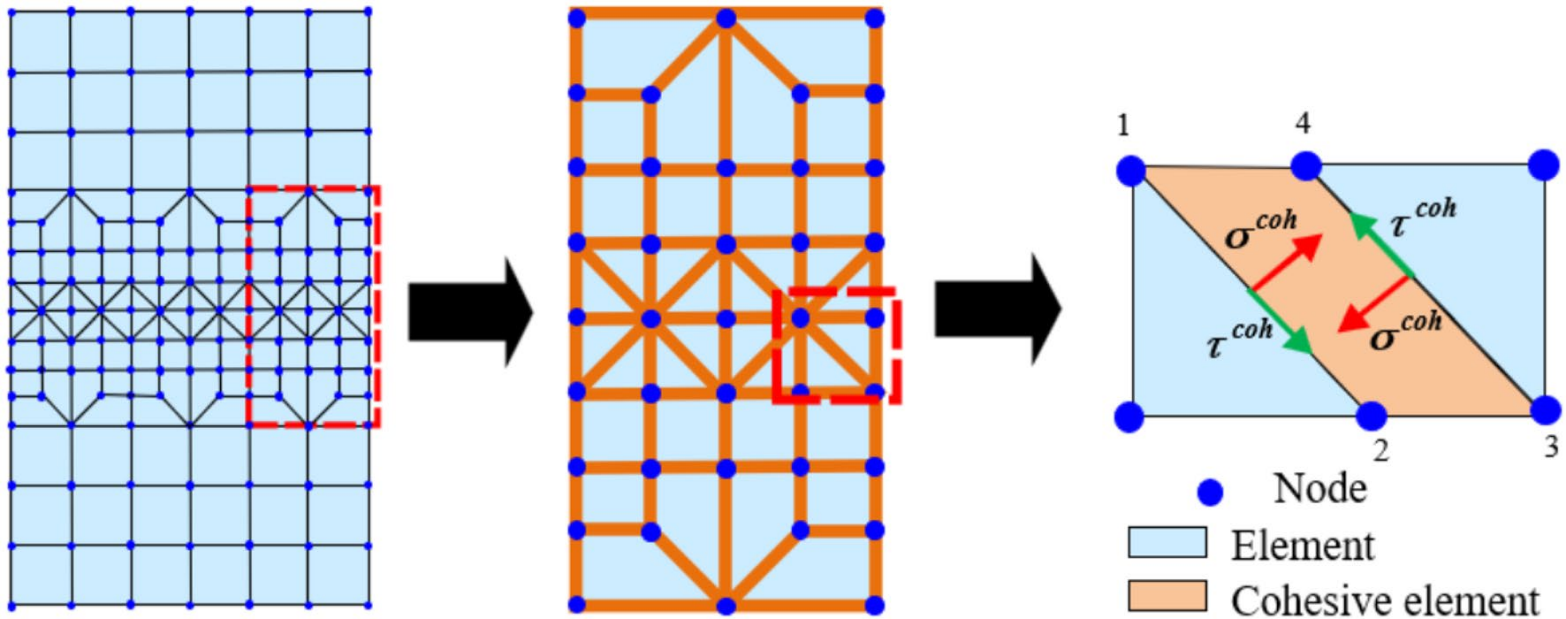
Schematic diagram of discretization of computational model by 2D Hybrid Finite‑Discrete element method.

The HFDEM is developed based on principles from continuum mechanics, nonlinear fracture mechanics utilizing the cohesive-zone model (CZM), and contact mechanics. As illustrated in Fig. 3, unlike the conventional finite element method, the HFDEM introduces joint elements at the interface of the finite element to simulate the fracture behavior of the specimen. In the 2D HFDEM, the joint element is composed of two joint surfaces, left (which is consists of node 1 and node 2) and right (which is consists of node 3 and node 4) surface. The stress between the two joint surfaces is determined by the CZM.

### Cohesive zone model for crack propagation

Cohesive zone theory, also known as traction–separation laws (TSL), focuses on the relationship between interface stress and relative displacement, which adheres to a specific functional relationship termed the cohesive constitutive relation. All TSL models are phenomenological, meaning that the cohesive force-relative displacement curve is an approximate quantitative relationship that does not define or describe the actual fracture physical process. Common forms of TSL include polynomial, exponential, bilinear, and trapezoidal models. However, different forms of TSL do not significantly affect the analysis results^37^. The trapezoidal CZM is renowned for its effectiveness and versatility in capturing plastic deformation near crack zones, with the capability to transition to a bilinear form under specific parameter conditions. Consequently, this study employs the trapezoidal CZM to investigate crack propagation under loading conditions.

#### Under quasi-static loading

**Figure 4:**
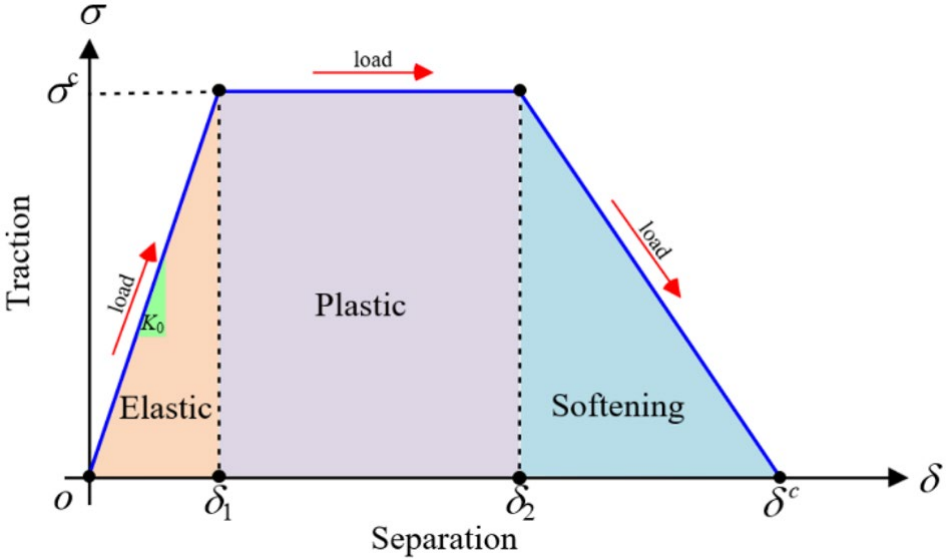
Schematic diagram of the trapezoidal traction separation law under quasi-static loading.

The trapezoidal TSL, depicted in Fig. 4, primarily consists of four shape parameters (*σ*^*c*^, *δ*_1_, *δ*_2_, and *δ*^*c*^), where *σ*^*c*^ is the critical cohesive traction, *δ*_1_ is the separation at which the deformation become permanent, *δ*_2_ is the separation at which the cohesive element softening is assumed to start, and *δ*^*c*^ is the critical cohesive separation caused by the failure of cohesive elements.

The standard trapezoidal CZM can be mathematically represented as:

1 $$ \sigma = \left\{ \begin{gathered} {K_o} \cdot \delta \quad \quad \quad \quad \quad if{\kern 1pt} {\kern 1pt} {\kern 1pt} \delta < {\delta_1} \hfill \\ {\sigma^c}\quad \quad \quad \quad \quad \quad if{\kern 1pt} {\kern 1pt} {\delta_1} \leqslant \delta < {\delta_2} \hfill \\ \frac{{{\delta^c} - \delta }}{{{\delta^c} - {\delta_2}}} \cdot {\sigma^c}\quad {\kern 1pt} {\kern 1pt} {\kern 1pt} \quad if{\kern 1pt} {\kern 1pt} {\delta_2} \leqslant \delta < {\delta^c} \hfill \\ 0\quad {\kern 1pt} {\kern 1pt} \quad {\kern 1pt} {\kern 1pt} \quad {\kern 1pt} {\kern 1pt} \quad {\kern 1pt} {\kern 1pt} \quad {\kern 1pt} {\kern 1pt} {\kern 1pt} \,\,{\kern 1pt} if{\kern 1pt} {\kern 1pt} {\delta^c} \leqslant \delta \hfill \\ \end{gathered} \right. $$

2 $$ \left\{ \begin{gathered} {\delta_1} = \frac{{\sigma^c}}{{K_o}} \hfill \\ {\delta_2} = \alpha \cdot {\delta^c} \hfill \\ \end{gathered} \right. $$

where *K*_0_ is the cohesive stiffness of a damaged cohesive element, *α* is a constant parameter, and its value can be set to 0.75^38^. The area enclosed by the traction–separation curve represents the total dissipated energy (*G*^c^), which can be mathematically represented as:

3 $${G^c} = \frac{{\sigma^c}}{2}\left( {{\delta^c} - {\delta_1} + {\delta_2}} \right)  $$

#### Under cyclic dynamic loading

Using traditional CZM to simulate the damage characteristics of rocks under cyclic dynamic loads erroneously indicates that the rocks remain undamaged. This contradicts experimental evidence showing that rocks exhibit fatigue damage when subjected to cyclic dynamic loads below their monotonic strength. Such fatigue damage gradually accumulates with an increasing number of cycles, eventually leading to the failure of the cohesive element^39^. Therefore, to effectively simulate fatigue crack propagation with the CZM, cyclic loading damage must be considered.

**Figure 5:**
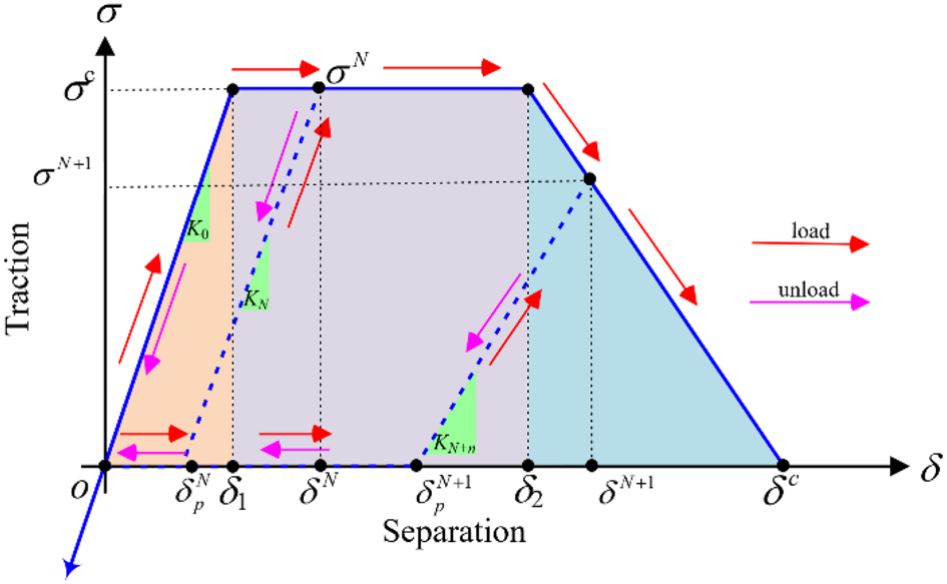
Schematic diagram of the trapezoidal traction separation law under cyclic dynamic loading.

Figure 5 illustrates a trapezoidal CZM under cyclic dynamic loading, the parameter *K*_*N*_ represents the cohesive stiffness after *N* cycles loading. ${\delta^N}$,${\sigma^N}$, and $\delta_p^N$ respectively represent the maximum separation, corresponding traction, and accumulated plastic separation after *N* cycles of loading. ${\delta^{N + 1}}$,${\sigma^{N + 1}}$, and $\delta_p^{N + 1}$ respectively represent the maximum separation, corresponding traction, and accumulated plastic separation after *N* + 1 cycles of loading.

The trapezoidal CZM under cyclic dynamic loading can be mathematically represented as:

4 $$ \sigma = \left\{ {\begin{array}{*{20}{c}} {{K_o} \cdot \delta \quad \quad \quad \quad \quad \quad \quad \quad \quad \quad \quad \quad \quad if{\kern 1pt} {\kern 1pt} {\kern 1pt} \delta < 0} \\ {\left( {\frac{{\sigma^N}}{{{\delta^N} - \delta_P^N}}} \right)\left( {\delta - \delta_P^N} \right)H\left( {\delta - \delta_P^N} \right)\quad \quad if{\kern 1pt} {\kern 1pt} 0 \leqslant \delta \leqslant {\delta^N}} \\ {{\sigma^c}\quad \quad \quad \quad \quad \quad \quad \quad \quad \quad \quad \quad \quad \quad if{\kern 1pt} {\kern 1pt} {\delta_1} \leqslant \delta < {\delta_2}} \\ {\left( {1 - D\left( \delta \right)} \right) \cdot {\sigma^c}\quad {\kern 1pt} {\kern 1pt} \quad \quad \quad \quad \quad \quad \quad \quad {\kern 1pt} if{\kern 1pt} {\kern 1pt} {\delta_2} \leqslant \delta < {\delta^c}} \\ {0\quad {\kern 1pt} {\kern 1pt} \quad {\kern 1pt} {\kern 1pt} \quad {\kern 1pt} {\kern 1pt} \quad {\kern 1pt} {\kern 1pt} \quad {\kern 1pt} {\kern 1pt} {\kern 1pt} \,\,\quad \quad \quad \quad \quad \quad \quad \quad {\kern 1pt} if{\kern 1pt} {\kern 1pt} {\delta^c} \leqslant \delta } \end{array}} \right. $$

5 $$ H\left( {\delta - \delta_P^N} \right) = \left\{ {\begin{array}{*{20}{c}} {1\;\;\;\;\;\;\delta - \delta_P^N > 0} \\ {0\;\;\;\;\;\delta - \delta_P^N < 0} \end{array}} \right. $$

6 $$ D\left( \delta \right) = 1 - \frac{{{\delta^c} - \delta }}{{{\delta^c} - {\delta_2}}}{\kern 1pt} \quad \quad if{\kern 1pt} {\kern 1pt} {\delta_2} \leqslant \delta < {\delta^c} $$

7 $$ \delta = \delta_p^N + {\delta^{cyc}} $$

8 $$ \delta_p^{N + 1} \approx \delta_p^N + \frac{{{\delta^N} - \delta_p^N}}{\beta } $$

where ${\delta^{cyc}}$ represents the cyclic separation resulting from the applied load at any time increment. *β* is a material parameter that characterizes the degree of plastic separation^38^, and its value can be set to 1.5.

Then, the increase in accumulated dissipated energy can be expressed as follows:

9 $$ \Delta G = \left\{ \begin{gathered} \frac{{{\sigma^c}\left( {{\delta^N} + \delta_P^N - {\delta_1}} \right)}}{2}\;\;\;\;\;\;\;\;\;\;\;\;\;\;\;\;\;\;\;\;\;\;\;\;\;\;\;\;if{\kern 1pt} {\kern 1pt} {\delta^N} \leqslant {\delta_2} \hfill \\ \frac{{{\sigma^c}\left( {{\delta^N} + {\delta_2} - {\delta_1}} \right) - {\delta^N}\left( {{\delta_2} - \delta_P^N} \right)}}{2}\;\;\;\;\;\;if{\kern 1pt} {\kern 1pt} {\delta^N} > {\delta_2} \hfill \\ \end{gathered} \right. $$

Drawing on the rate-dependent cohesive model concept introduced by Salih et al.^40^, the expression for frequency dependent critical stress can be mathematically expressed as:

10 $$ {\sigma^c}\left( f \right) = \sigma_q^c \cdot {e^{\left( { - \frac{{f_0}}{f}} \right)}} $$

where $\sigma_q^c$ is the quasi-static critical traction. *f*_0_ is the base frequency, and can be set as 0.05 Hz.

The cohesive traction force mentioned above is mainly normal stress. In this study, the shear stress also satisfies the trapezoidal CZM, but it is 10 times the numerical value of the shear stress. Tensile crack occurs when cohesive force model fails under normal stress; when it fails under shearing stress, a shear crack is produced. When the normal stress and shear stress fail together, the resulting crack is a mixed crack.

### Calculation of contact forces between discontinuous bodies

**Figure 6:**
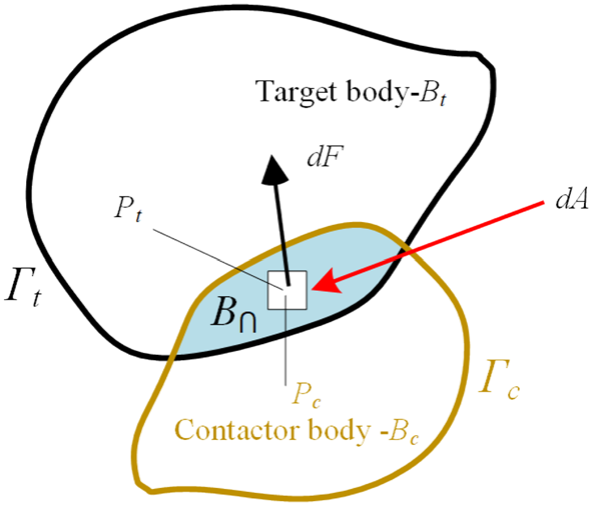
The contact force of the potential at the contact overlap between the target body and the target body.

As shown in Fig. 6, if the joint element fails, the model transition from continuous to discontinuous, then contact interaction between discontinuous bodies generates contact force. *B*_*c*_ is target body, *B*_*t*_ is contactor body, *B*_⋂_ is the overlapping area of *B*_*c*_ and *B*_*t*_. *Г*_*Bt*+*Bc*_ is the overlapping boundaries of *B*_*c*_ and *B*_*t*_. *d*_*A*_ is size of the infinitesimal overlap, then the contact forces over the overlapping area can be written as:

11 $$ {f_c} = {P_{n\_con}}\oint_{{\Gamma_{Bt + Bc}}} {\left( {{\varphi_c} - {\varphi_t}} \right){n_\Gamma }d\Gamma }  $$

where, *φ*_*t*_ the potential function of *B*_*t*_, *φ*_*c*_ the potential function of *B*_*c*_, *P*_*n_con*_ is the normal penalty of the contact surface, *n*_Г_ is the outward unit normal to the boundary of the overlapping area.

The frictional force in the tangential direction is computed based on the friction coefficient of the contact surface, normal contact force, and shear penalty parameters^32^:

12 $$ \tau { = }\frac{{v_r}}{{\left| {v_r} \right|}}\min imum\left( {{P_{t\_con}}h\left| {\delta_s} \right|,\;\tan \left( \varphi \right){\sigma_{n\_con}}} \right) $$

where, *P*_*t_con*_ is the tangential penalty of the contact surface, *v*_*r*_ is the projection vector of the relative velocity of the contact surface on the contact surface, *σ*_*n_con*_ is the normal contact force on the contact surface. *δ*_*s*_ is the cumulative distance of relative shear sliding of the surface from contact, which can be computed based on the time steps and relative shear sliding speed.

### Finite element explicit dynamic equation

Substituting the above formulas into Eq. (13), the hybrid finite element discrete element method can be solved based on the explicit dynamic finite element calculation principle.

13 $$ M\ddot u\left( t \right) + C\dot u\left( t \right) - {F_{\operatorname{int} }}\left( t \right) - {F_{ext}}\left( t \right) - {F_{coh}}\left( t \right) = 0 $$

where, *M* is the mass matrix and *C* is the damping matrix, *ü*(*t*) is the acceleration vector of the node, *C* is the damping matrix,$\dot u\left( t \right)$ is velocity vector of the node, *F*_int_(*t*) is the internal force of the model (e.g. Element stress and nodal forces), *F*_ext_(*t*) is external load (mainly referring to the load applied by the model boundary), *F*_coh_(*t*) is the contact force (e.g. the contact force between discontinuous boundaries of the element). Therefore, from a theoretical perspective, the HFDEM can be considered as an extension of the FEM method. The HFDEM simulation encompasses intricate physical interactions and the management of nonlinear problems. CUDA C is particularly adept at handling these complexities, thereby enhancing the practicality of the simulation. Consequently, this article employs CUDA C to implement the entire computational process of HFDEM.

## Testing and validation of hybrid finite-discrete element simulation method

### Sample preparation and parameters testing

The green sandstone samples used in the laboratory uniaxial compression tests were obtained from a quarry located in Luzhou City, Sichuan Province, China. These samples are sedimentary rocks that have undergone weathering, erosion, transportation, and deposition in a basin, with siliceous cementation. To avoid differences in test results due to different sample sizes, the sample was cut into blocks 120 mm high, 60 mm wide and 60 mm thick, which is complies with the recommended values by the International Society for Rock Mechanics.

The MTS815 rock mechanics test system was employed for the tests. Before the test, lubricating oil was applied to the end of the sandstone specimen, which was then placed between the rigid pressure head and the base of the test system, all of the specimens were conducted in displacement-controlled mode. The basic mechanical parameters of the standard samples were determined through conventional uniaxial compression, triaxial, and Brazilian splitting tests. The uniaxial compressive strength of the standard samples is 49.14 MPa, other mechanical parameters are listed in Table 1.

**Table 1:** Material properties.

Properties	Density, *ρ* (kg/m^3^)	Young’s modulus, *E* (GPa)	Poisson’s ration, *υ*	Tensile strength, *Ts* (MPa)	Cohesion, *c* (MPa)	Internal friction angle, *φ* (°)
Value	2300	5.43	0.21	4.91	16.21	27

**Figure 7:**
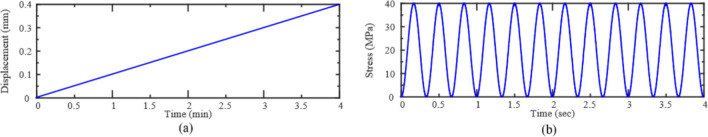
Schematic diagrams illustrating the four loading paths used by the laboratory experiments. (**a**) Uniaxial compression path, (**b**) cyclic dynamic loading with frequency of 3.0 Hz.

Four different loading paths were used for the tests, a uniaxial compression path and a cyclic loading path (as depicted in Fig. 7). The uniaxial compression path with an average loading rate of 0.1 mm/min, which can be considered as a quasi-static loading, the loading was stopped when the sample failed.

In the dynamic loading process, the specimens were subjected to cyclic loading with an amplitude of 40 Mpa and frequencies of 3.0 Hz until failure.

### Calibration of model parameters and numerical models for simulation

There are a total of 12 parameters that need to be calibrated, among them, density of mass, Young’s modulus, Poisson’s ration, Tensile strength, Cohesion and Internal friction angle can be obtained through conventional rock mechanics parameter testing experiments. Porosity can be determined by nuclear magnetic resonance instrument. *δ*_1_ and *δ*^c^ can be obtained by load–displacement curves. The normal contact penalty and tangent contact penalty have the same value, and the value of fracture penalty is 10 times the normal contact penalty. By calculating the compressive strength of rock samples with different penalty values through uniaxial compression tests under quasi-static loads, until it is consistent with the laboratory test results.

**Figure 8:**
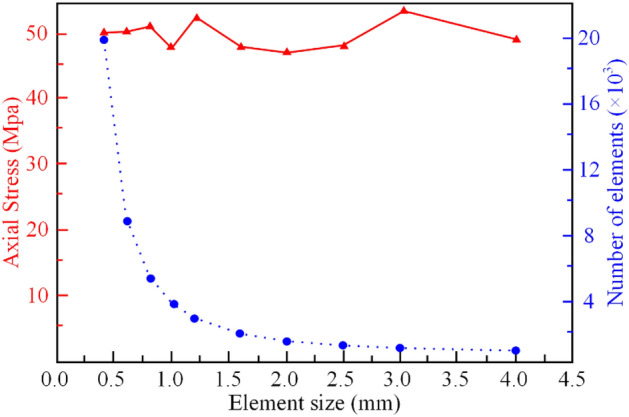
Uniaxial compressive strength with different Elements sizes.

To verify the reliability of numerical simulation using the HFDEM, the model with rectangular specimen (60 mm × 120 mm) is selected as a typical case, the material parameters of the model are listed in Table 2, and displacement loading at a loading rate of 0.05 m/s was applied to satisfy quasi-static loading conditions. To accurately simulate the crack propagation process, it is often necessary to minimize the size of grid elements. However, it leads to increased computational complexity. Figure 8 illustrates the changes in uniaxial compressive strength observed in rectangular specimens under quasi-static loading conditions following their discretization into triangular mesh elements varying in size from 0.5 to 4.0 mm. The results indicate that when the discrete element size ranges from 0.5 to 1.2 mm, the uniaxial compressive strength of the rock sample obtained through numerical simulation closely matches the experimental value. As the grid size increases from 1.2 to 4.0 mm, the uniaxial compressive strength does not show any significant increase or decrease, remaining consistently close to the experimental value. However, the fluctuation amplitude becomes relatively large, with the maximum deviation reaching approximately 5 MPa. In order to balance calculation accuracy and efficiency, the element size can be set to 8‰ to 8% of the shortest side length of the model. Then the model is discrete by triangular elements with a side length of approximately 0.5 mm.

**Table 2:** Material properties of numerical study.

Properties	Numerical study
Density, *ρ* (kg/m^3^)	2300
Young’s modulus, *E* (GPa)	5.43
Poisson’s ration, *υ*	0.21
Tensile strength, *Ts* (MPa)	4.91
Cohesion, *c* (MPa)	16.21
Internal friction angle, *φ* (°)	27
Porosity, *e* (%)	4
*δ*_1_ (mm)	0.12
*δ*^c^ (mm)	1.2
Normal contact penalty, *P*_n_con_ (GPa/m)	543
Tangent contact penalty, *P*_*ta*n_con_ (GPa/m)	543
Fracture penalty, P_*f*_, P_*tan*_, P_*overlap*_ (GPa/m)	5430

### Comparison of indoor test and numerical simulation results

#### Under quasi-static loading

**Figure 9:**
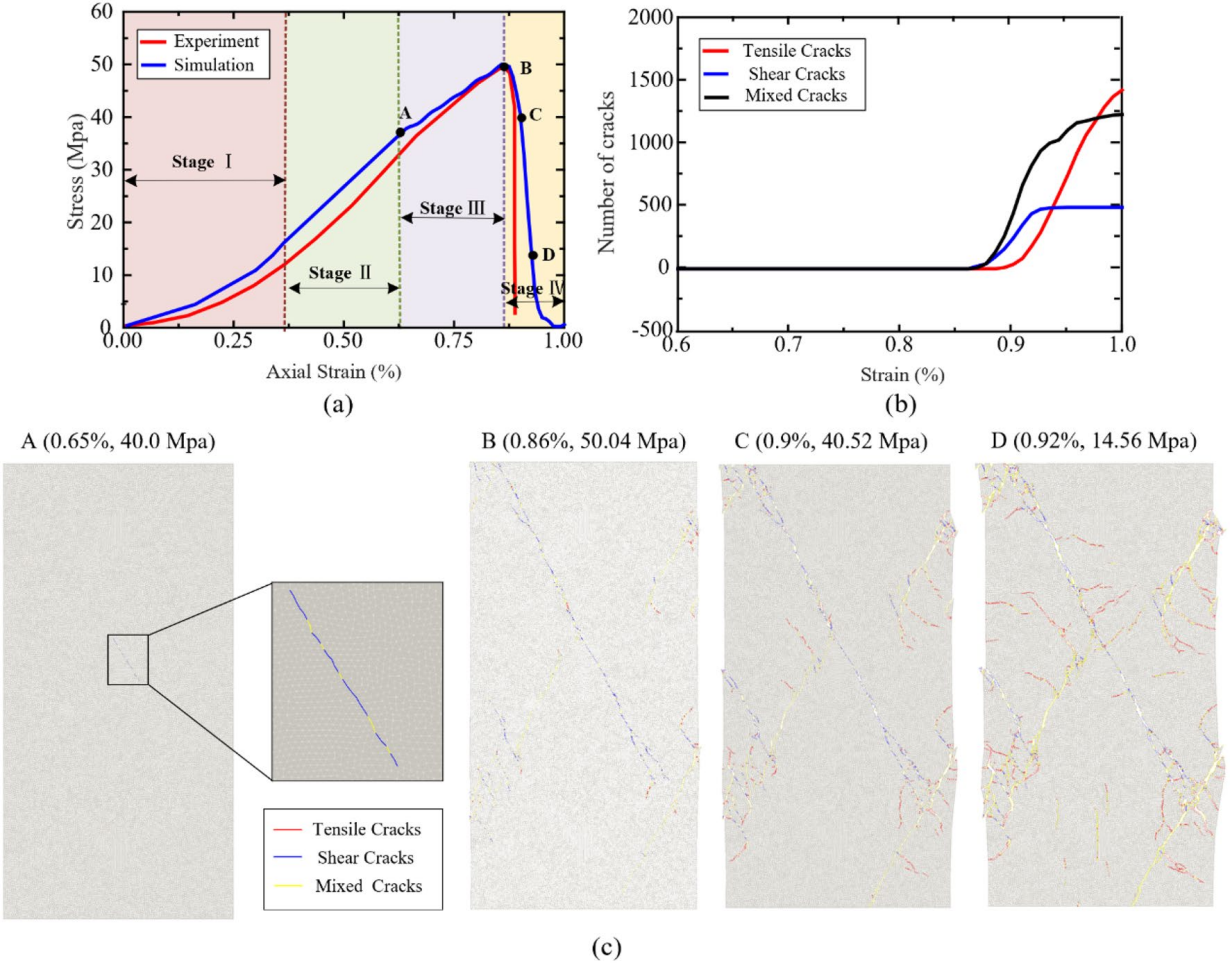
(**a**) Comparison of stress–strain curves obtained from laboratory tests and numerical simulations under quasi-static loads. (**b**) Curve of crack quantity under different strains under quasi-static uniaxial tests simulated by HFDEM. (**c**) Distribution of different types of cracks in rock samples under different strain states derived from the HFDEM numerical results (Tensile cracks are shown in red color. Shear cracks are shown in blue color. Mixed cracks are shown in yellow color).

The stress–strain curves obtained from laboratory tests and numerical simulations under quasi-static loading conditions are presented in Fig. 9a. It can be observed that, the stress–strain curves obtained from laboratory tests can be divided into four stages: the compaction stage, the elastic deformation stage, the unstable development stage, and the post-failure stage. During the compaction stage, the stress–strain curve exhibits a slight upward bending due to the closure of some microcracks within the specimens under initial compressive stress. This phenomenon is attributed to the presence of microcracks that are initially closed and gradually open as the stress increases. In the elastic deformation stage, the stress–strain curve shows a nearly linear growth trend. The rock specimen behaves elastically, with the stress increasing proportionally to the strain during this stage. As the axial pressure continues to increase, new microcracks initiate during the nonelastic deformation stage of the specimens. This leads to a deceleration in the growth rate of the stress–strain curve, resulting in a downward bending phenomenon. In this stage, as the strain increases, new cracks within the rock sample continue to develop and coalesce, leading to a rapid decrease in the stress–strain curve after reaching its peak (i.e., uniaxial compressive strength). After reaching the uniaxial compressive strength, the specimens enter the post-failure stage. The stress–strain curve exhibits a declining trend as the material experiences further damage and deformation.

Figure 9b shows the Curve of crack quantity under different strains under quasi-static uniaxial tests simulated by HFDEM. In the numerical simulation, the uniform solid medium is assumed in the numerical model, and the linear elastic constitutive model is adopted. The stress–strain curves also exhibit four stages: the compaction stage, elastic deformation, nonelastic deformation, and post-failure stages. The stress–strain curves obtained from the numerical simulations and laboratory experiments show good agreement in the nonelastic deformation and post-failure stages. When the strain is 0.86%, the rock sample reached its ultimate compressive strength under uniaxial compression conditions, and cracks were beginning to appear in the rock sample, mainly shear cracks. As the strain gradually increased, the three types of cracks also gradually increased. After the strain reached 0.92%, the number of shear cracks no longer increased, while the other two types of cracks continued to increase. Among them, the growth rate of tensile cracks was greater than that of mixed cracks.

Figure 9c presents the distribution of different types of cracks in rock samples, as obtained from HFDEM numerical results at four strain points: 0.65%, 0.86%, 0.9%, and 0.92%. It can be observed that when the strain ranged from 0.65% to 0.92%, the rock sample completed the process of crack initiation, propagation, and coalescence. On the primary fracture plane, shear cracks are predominant, while tensile and mixed cracks are predominant on the secondary fracture plane. The final failure mode of the rock sample is characterized by X-type conjugate shear plane shear failure.

#### Under cyclic dynamic loading

**Figure 10:**
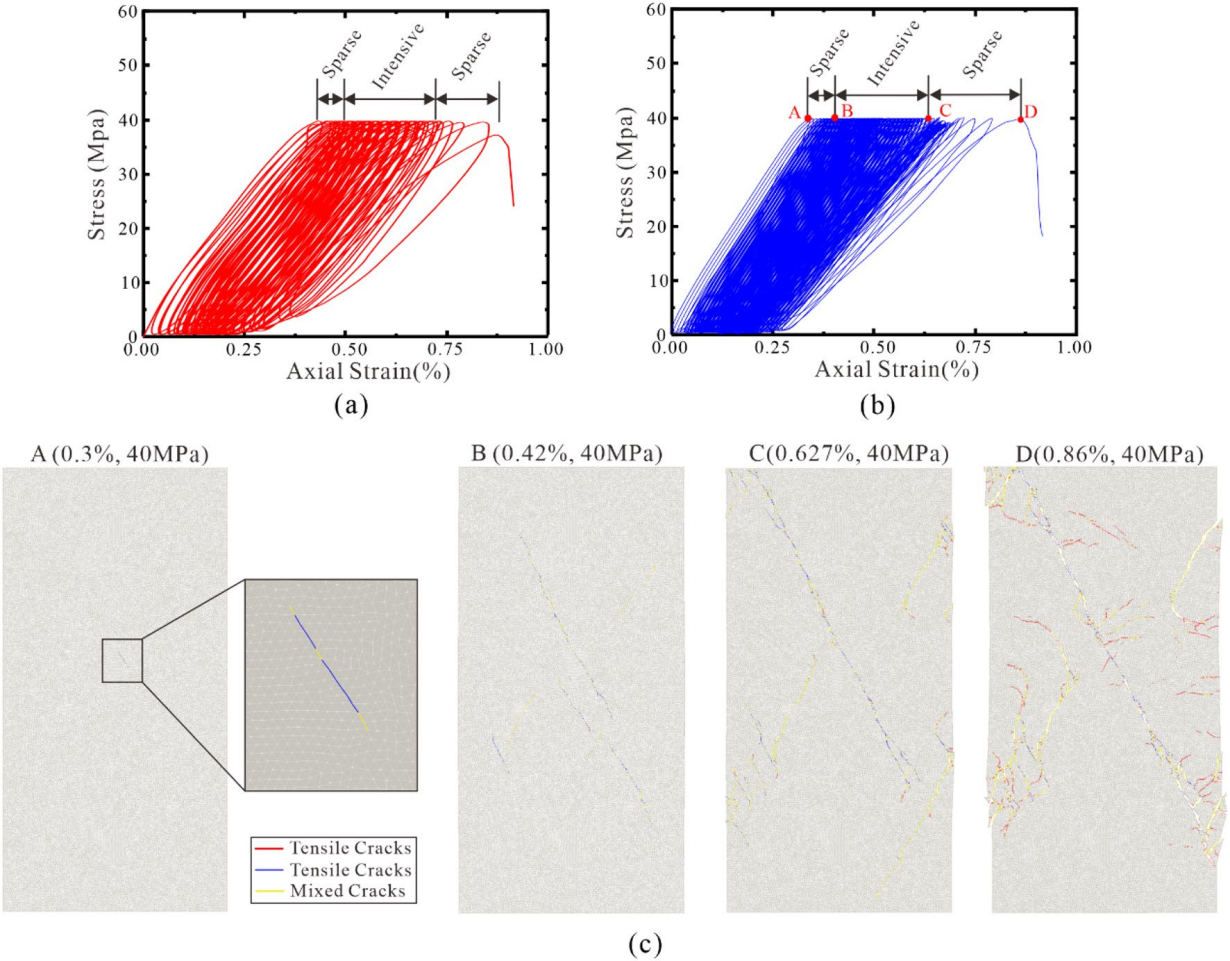
(**a**) Stress–strain curves derived from uniaxial compression test under cyclic dynamic load (**b**) Stress–strain curves derived from numerical simulations under cyclic dynamic load. (**c**) Distribution of different types of cracks in rock samples under different strain states derived from the HFDEM numerical results (Tensile cracks are shown in red color. Shear cracks are shown in blue color. Mixed cracks are shown in yellow color).

Figure 10a,b respectively present the stress–strain curves obtained from laboratory tests and numerical simulations under cyclic dynamic loading conditions with an amplitude of 40 Mpa and frequencies of 3.0 Hz. During the entire loading process, the stress–strain curve can be divided into three stages: Stage I (sparse), Stage II (intensive), and Stage III (sparse).

Stage I represents the initial deformation phase under cyclic dynamic loading. During this stage, the closure of pre-existing cracks in the rock sample results in a rapid and irreversible increase in axial deformation. Notably, the plastic hysteresis loops are characterized by relatively large areas, and the loops are spaced relatively far apart. Throughout the loading process, the specimen absorbs a significant amount of energy, leading to pronounced hardening of the rock sample. This stage is relatively short in duration.

Stage II is characterized by a lower level of deformation under cyclic loading. Compared to the initial deformation stage, the plastic hysteresis loop area in this stage is smaller and tends to stabilize, while both the axial deformation and irreversible deformation per loading cycle remain relatively consistent. The energy absorbed by the specimen stabilizes, and the strain hardening exhibits a weakening trend. As illustrated in Fig. 10c, the pronounced influence of the strain hysteresis effect reduces the frequency of internal crack propagation within the specimen, significantly decreasing the strain growth rate, which directly impacts the fatigue life of the rock.

Stage III is the cycle acceleration deformation stage. During this stage, the area of the plastic hysteresis loop increases once more, leading to a significant rise in both axial and irreversible deformation. The strain rate also increases substantially compared to the previous two stages, and microcracks continue to propagate. Eventually, as internal cracks in the specimen coalesce, the specimen loses its structural integrity and rapidly fails.

The development pattern of the hysteresis loops in the specimen throughout the entire fatigue process exhibits a sparse-dense-sparse sequence. The gradual decrease in the slope of the hysteresis loops indicates a reduction in the elastic modulus of the rock sample as the number of loading cycles increases. The above comparative analysis shows that the HFDEM proposed in this article can accurately simulate the failure process of rocks under quasi-static and cyclic dynamic loading conditions.

## Models for uniaxial compression of specimens with prefabricated cracked flaw

**Figure 11:**
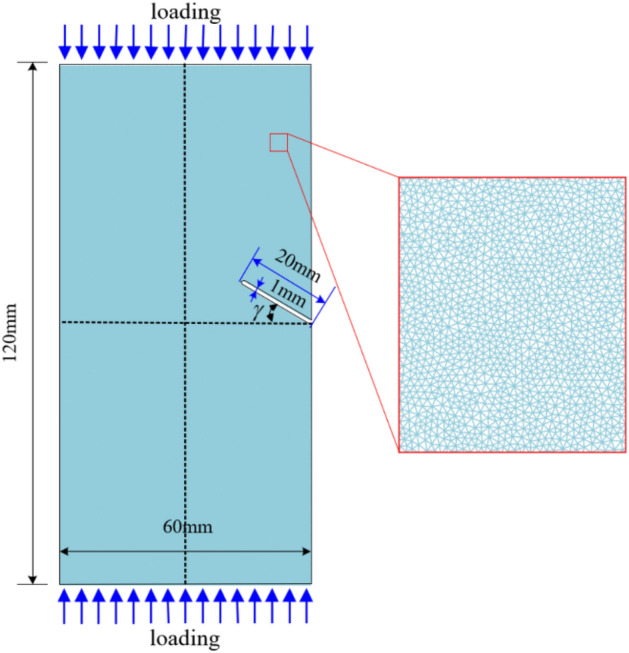
Two-dimensional numerical model of sandy mudstone specimens with an edge-flaw under quasi-static and dynamic loading.

**Figure 12:**
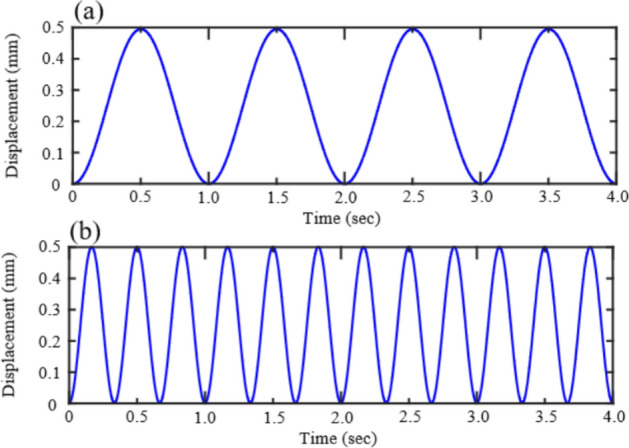
Schematic diagram of two cyclic dynamic loading paths used in numerical simulation, The two loading paths have the same amplitude and different frequencies, which are 1 Hz and 3 Hz respectively.

The geometric dimensions of the rock specimens are shown in Fig. 11. The pre-existing penetrating flaw is located in the middle of the right side of the specimen, with a size of 1 mm × 20 mm. The pre-existing flaw inclinations have been purposefully designed to be *γ* = 0°, 30°, 45°, and 60°. The material parameters of the model are listed in Table 2. Assuming the model is a uniform solid component (i.e., porosity set to 0), and the model is discrete by triangular elements with a side length of approximately 0.5 mm. Displacement loading at a loading rate of 0.05 mm/s was applied to satisfy quasi-static loading conditions. Considering that the actual seismic motion is composed of harmonic waves of different components, without loss of generality, this paper selects displacement time histories with amplitudes of 0.5 mm and frequencies of 1 Hz and 3 Hz as cyclic dynamic load inputs, as shown in Fig. 12.

### Under quasi-static loading

#### Strength characteristics

**Figure 13:**
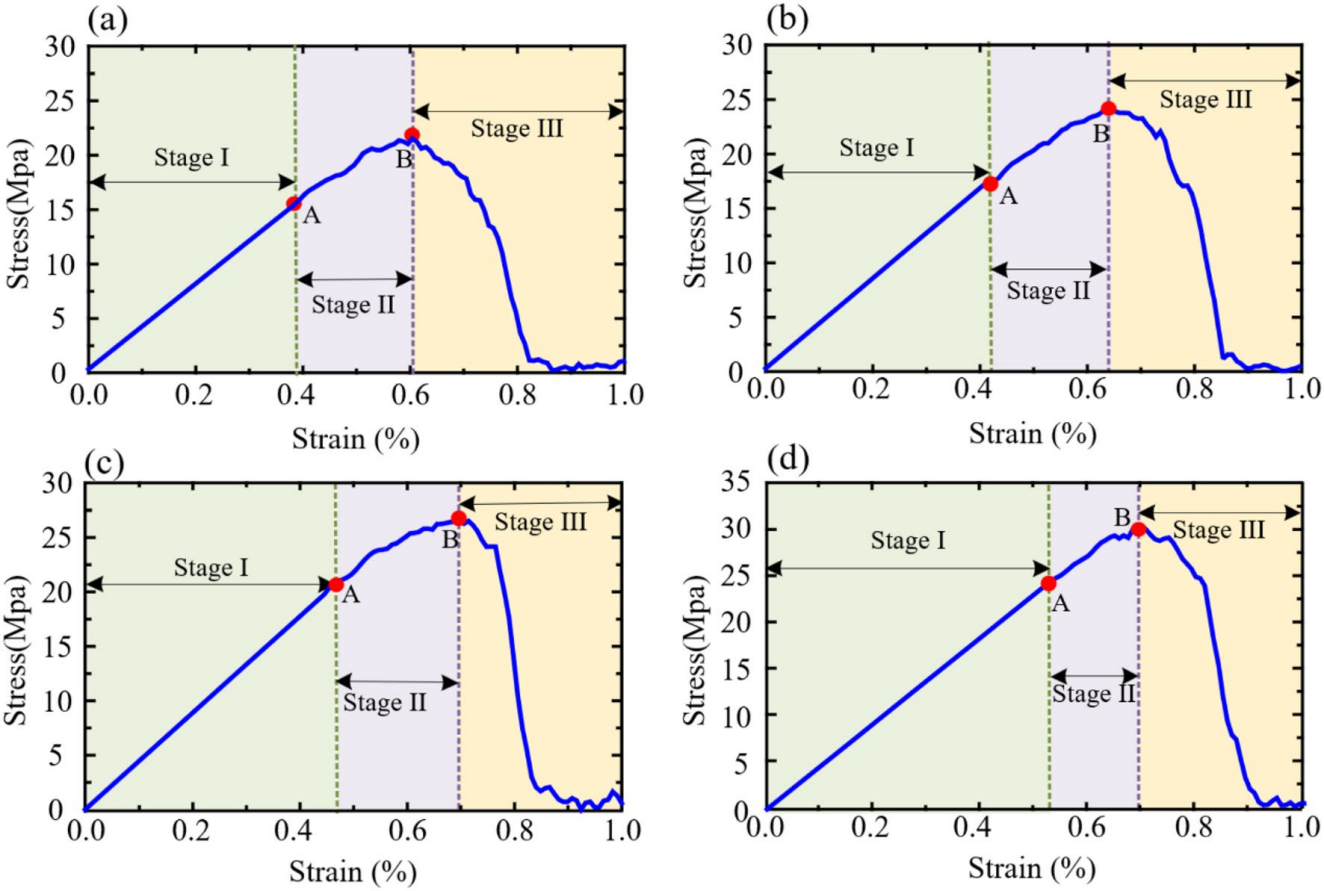
Stress–strain curves of different pre-existing flaw inclination models under quasi-static loading derived from the numerical simulation. (**a**) *γ* is 0°, (**b**) *γ* is 30°, (**c**) *γ* is 45°, and (**d**) *γ* is 60°

Figure 13 shows the stress–strain curves and crack quantity-strain curves of different pre-existing flaw inclination models under quasi-static loading derived from the numerical simulation. Due to the lack of consideration of porosity in the model, then the numerical model is assumed to be a mean solid single medium. Therefore, the stress–strain curve can be roughly divided into three stages: the elastic deformation stage (Stage I), the unstable development stage (Stage II), and the post-failure stage (Stage III). Point A and point B are the dividing points of the three stages respectively. It can be observed that with the increase of the inclination angle of the pre-existing flow, the strain energy stored in the sample gradually increases, and the strain difference between point B and point A gradually decreases. The slope of the stress–strain curve in the Stage I is the same, mainly because all numerical models use the same material and medium parameters.

**Figure 14:**
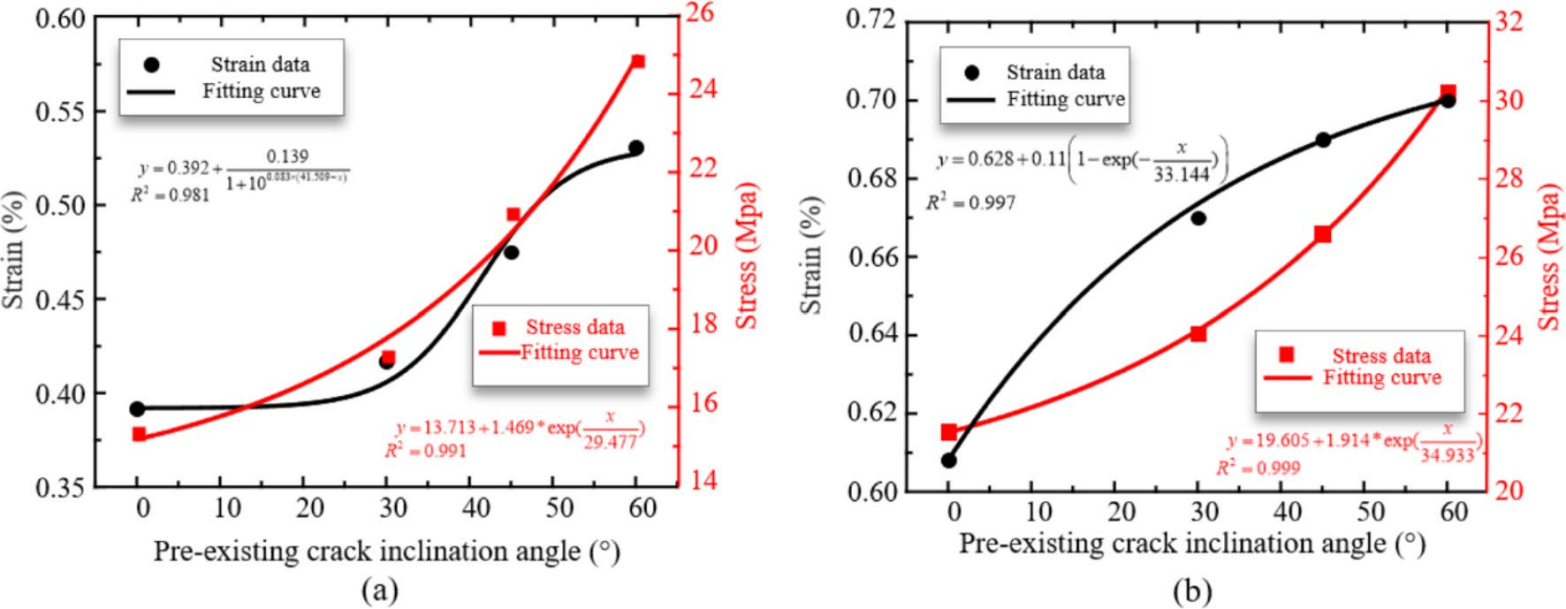
Stress and ultimate strain of rock samples with different pre-existing flaw angles under quasi-static loading. (**a**) Point A, (**b**) Point B.

Figure 14 shows the variation of stress and strain at points A and B in Fig. 12 with the inclination angle of pre-existing flow. Point A is the starting point for plastic deformation of the material. It can be observed that as the inclination angle of pre-existing flow increases, the stress and strain at point A increase exponentially, but the rate of stress growth first increases and then decreases. Point B is the end point of Stage II and the beginning point of Stage III, at which all models have peak stress. With the increase of pre-existing flow inclination Angle, the stress and strain at B point also increase exponentially.

In short, from the perspective of deformation characteristics and strength characteristics, the deformation characteristics of samples with small angle of edge-cut pre-existing flaw are more obvious under quasi-static loading.

#### Crack propagation characteristics

**Figure 15:**
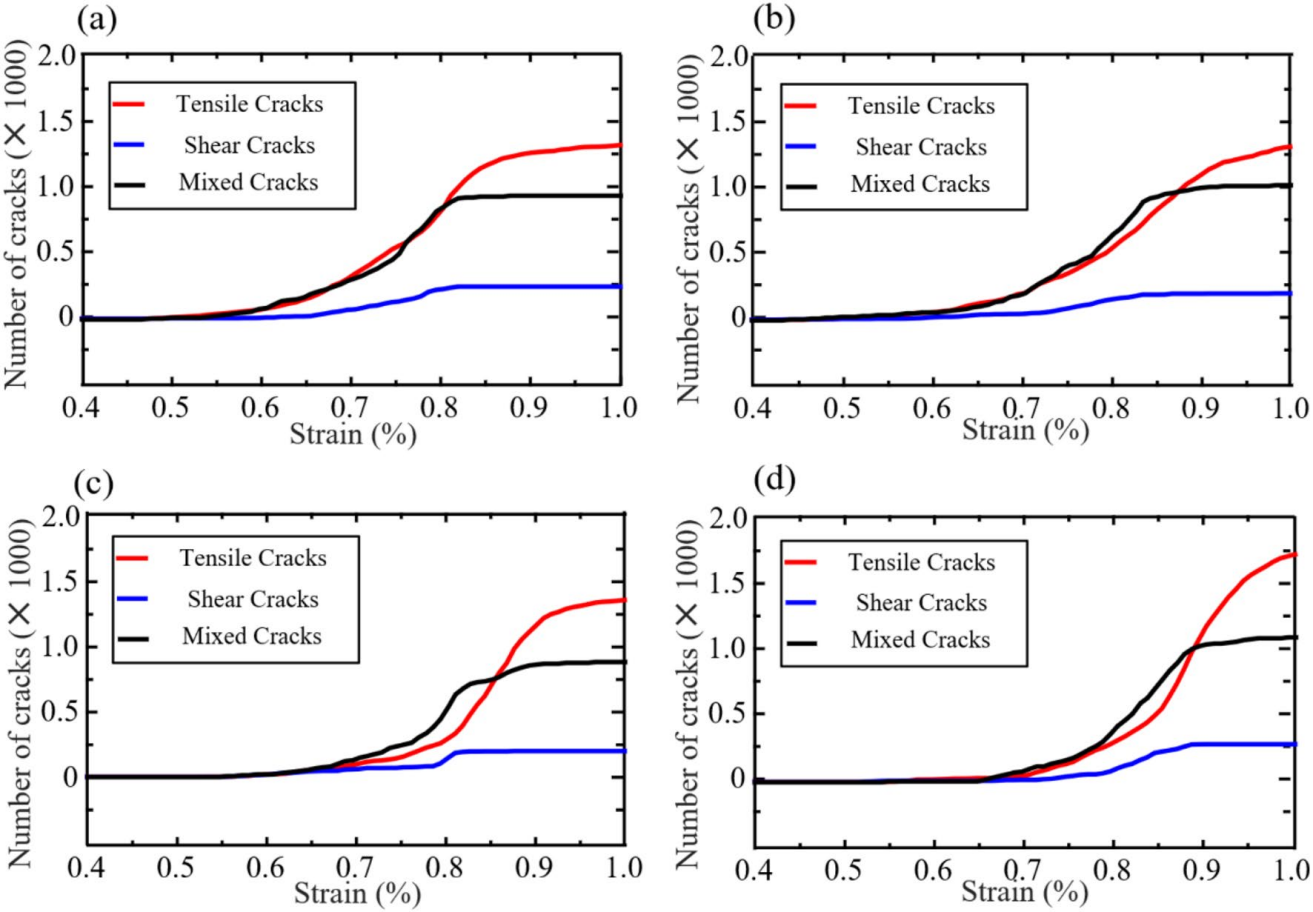
Crack quantity-strain curves of different pre-existing flaw inclination models under quasi-static loading derived from the numerical simulation. (**a**) *γ* is 0°, (**b**) *γ* is 30°, (**c**) *γ* is 45°, and (**d**) *γ* is 60°.

Figure 15 shows the crack quantity-strain curves of samples with different pre-existing flaw inclination within the strain range of 0.4–1.0%. The number of cracks in all specimens increases with the increase of strain. For the same specimen, the number of shear cracks is the smallest under the same strain conditions. In the case where *γ* = 0° under the same strain condition, the number of shear tension type cracks is the highest, followed by the number of mixed type cracks. In the case where *γ* is greater than 0° the number of mixed type cracks is the largest, followed by the number of tension type cracks. In the whole failure stage, the number of tensile cracks in all samples is more than that of the other two types. Therefore, under quasi-static load, *γ* mainly affects the crack in the plastic deformation stage.

**Figure 16:**
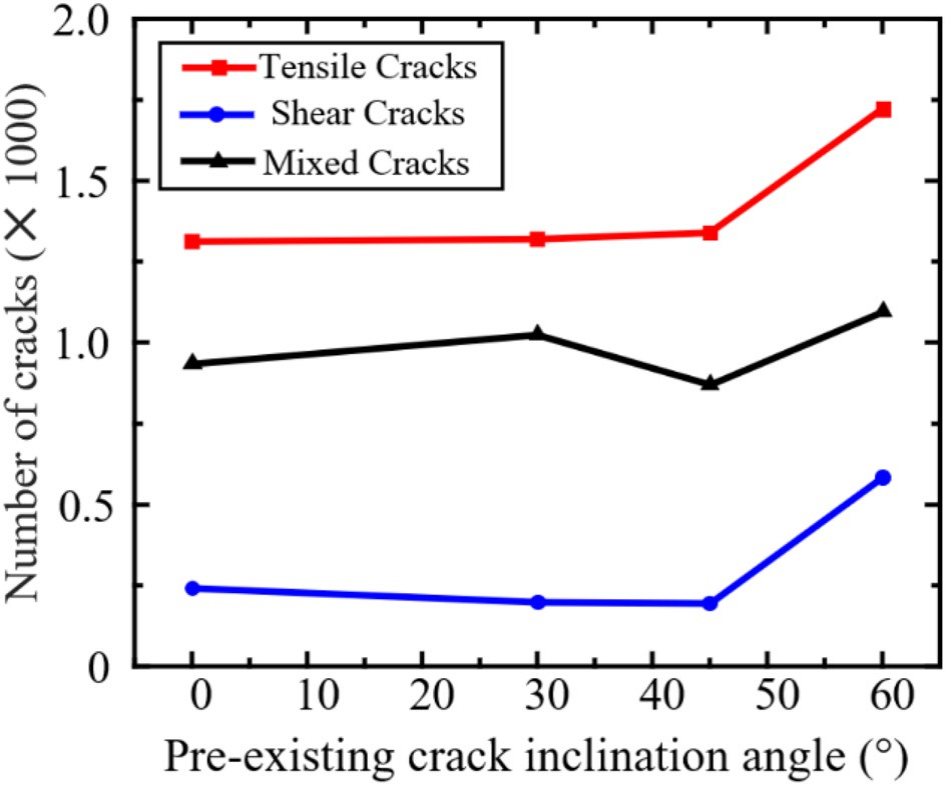
Number of cracks in specimens with different pre-existing flaw angles after failure.

Figure 16 illustrates the total number of cracks after failure under different joint inclination angles. Under quasi-static loading, the number of cracks in the specimen reaching the failure state increases with the increase of joint inclination angle. This phenomenon occurs because the specimen with *γ* = 60° has the highest bearing capacity and is also the most difficult to damage. However, at *γ* = 45°, there is a decreasing trend in mixed type cracks, mainly because at this angle, it is easier for the newly formed cracks at the tip of the prefabricated crack to slip.

#### Fracture mode of specimens

**Figure 17:**
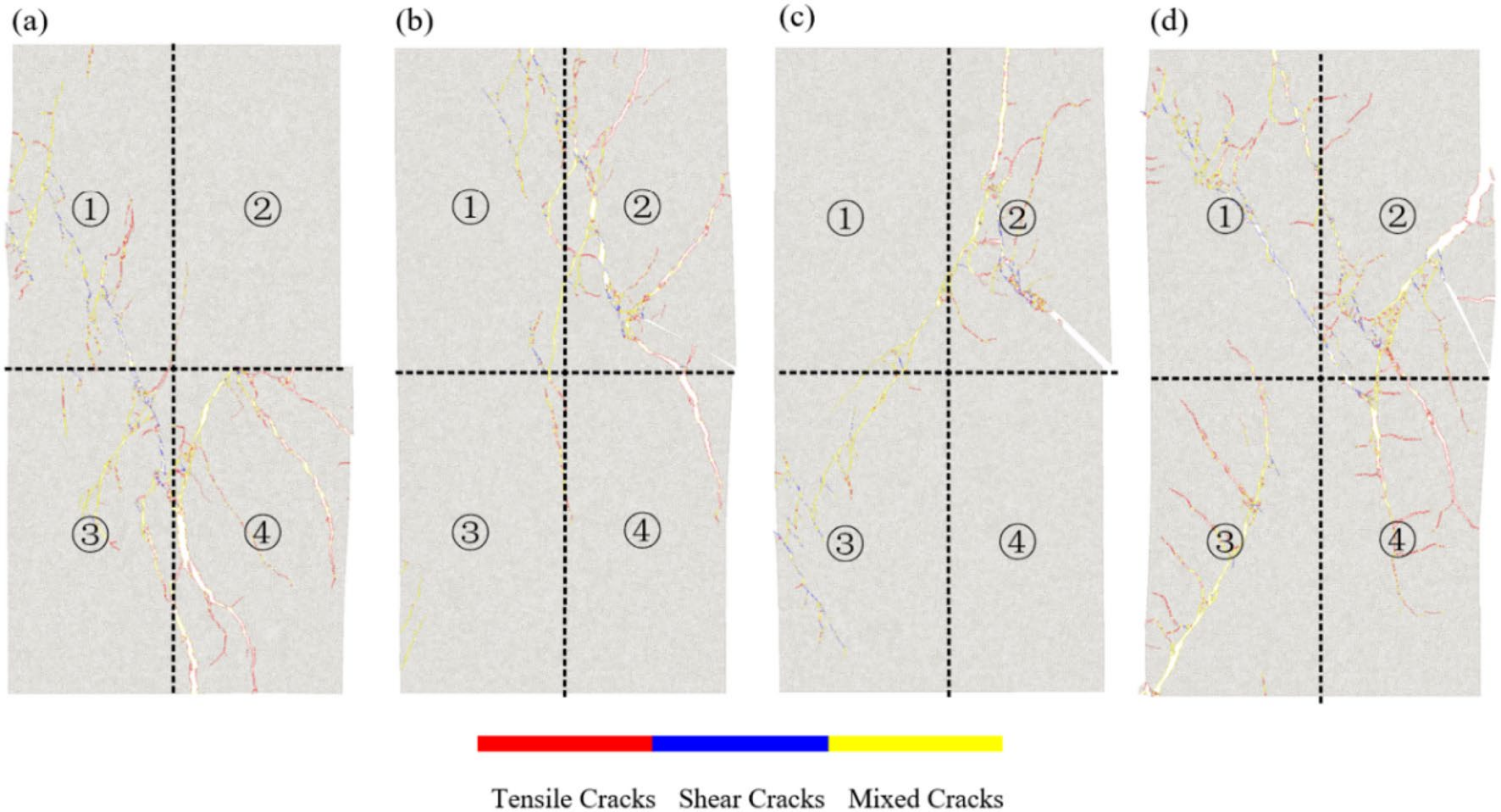
Failure modes of specimens with different inclination angles. (**a**) *γ* is 0°, (**b**) *γ* is 30°, (**c**) *γ* is 45°, and (**d**) *γ* is 60°.

Figure 17 shows that the failure modes of rocks with different pre-existing flaw angles are different under quasi-static loading. To facilitate analysis, the model is divided into four zones.

For the specimen with *γ* = 0°, which is mainly subjected to mixed type failure, but the forms of failure vary in different zones. There are variations in the pattern of destruction in different zones. The damage in zone 4 is the most serious, mainly tensile damage, and it is also the initial crack initiation zone. The crack then spread to zone 3 and finally to zone 1, and the main fissures are in blocks 1 and 4. The specimen with γ = 30° exhibits single shear failure mode, and the initial crack initiation occurred in zone 2, which was also the most severely damaged area. The main penetrating fissures was in zone 2 and Zone 4, and zone 3 was nearly intact. Double shear plane failure occurred in the specimen at *γ* = 45°, initial crack propagation mode is similar to that of the specimen with a γ = 30°, but when a local through fissures is formed in zone 2, the crack begins to propagate towards zone 3. The specimen with γ = 60° has the highest compressive strength and the highest degree of damage. The initial crack initiates in Zone 2 and then extends to Zone 4 to form a local through fissures.

In summary, the quasi-static loading has great influence on specimens with small inclination angle and low bearing capacity.

### Under cyclic dynamic loading

#### Strength characteristics

**Figure 18:**
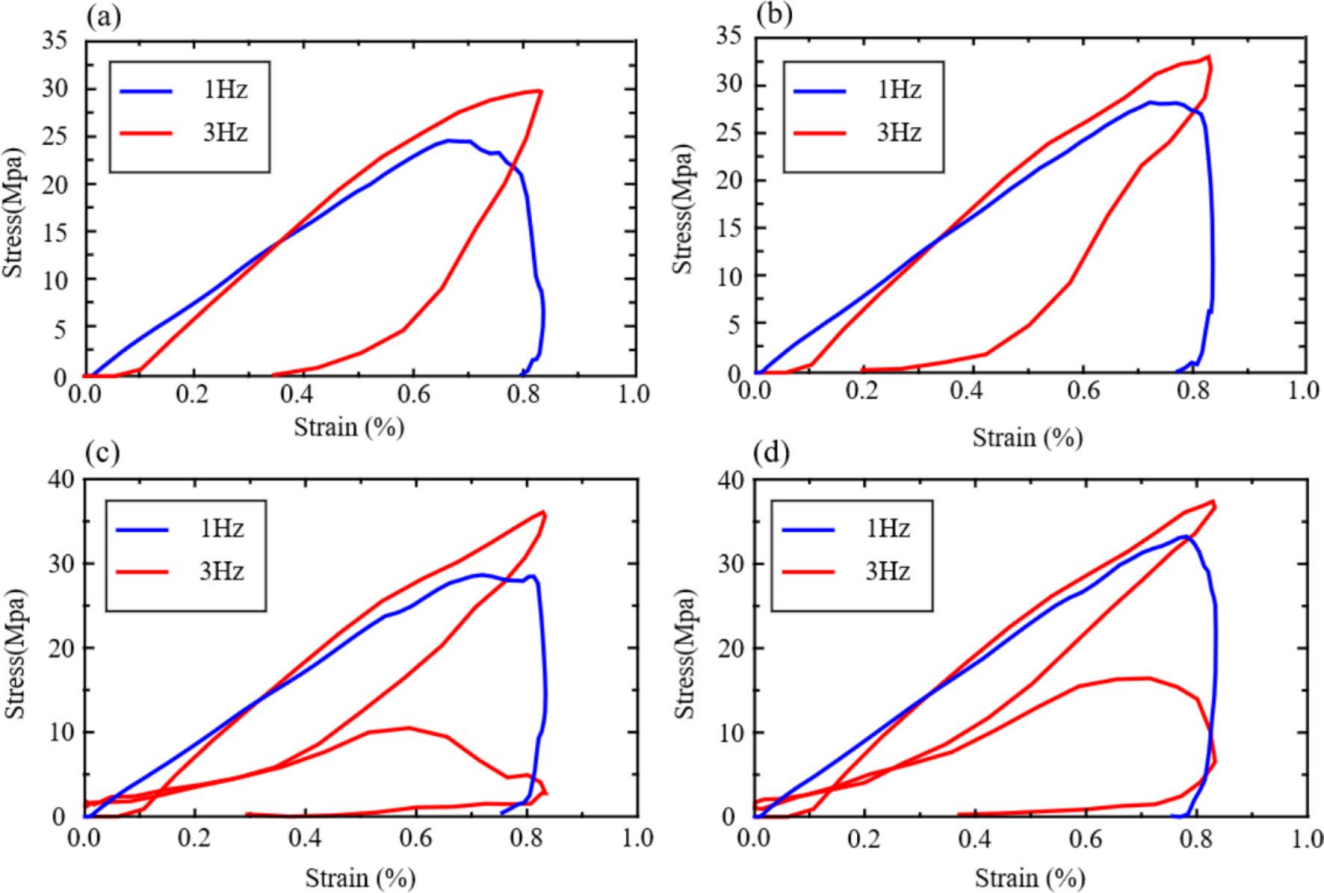
Stress–strain curves of different pre-existing flaw inclination models under cyclic-dynamic loading derived from the numerical simulation. (**a**) *γ* is 0°, (**b**) *γ* is 30°, (**c**) *γ* is 45°, and (**d**) *γ* is 60°

According to Fig. 18, it can be observed that under the dynamic cyclic load of 1 Hz, all rock samples undergo elastic deformation at the initial moment. However, under the dynamic load of 3 Hz, the rock sample undergoes instantaneous plastic deformation at the initial moment, and then enters the elastic deformation stage. Its dynamic elastic modulus increases with the increase of frequency.

**Figure 19:**
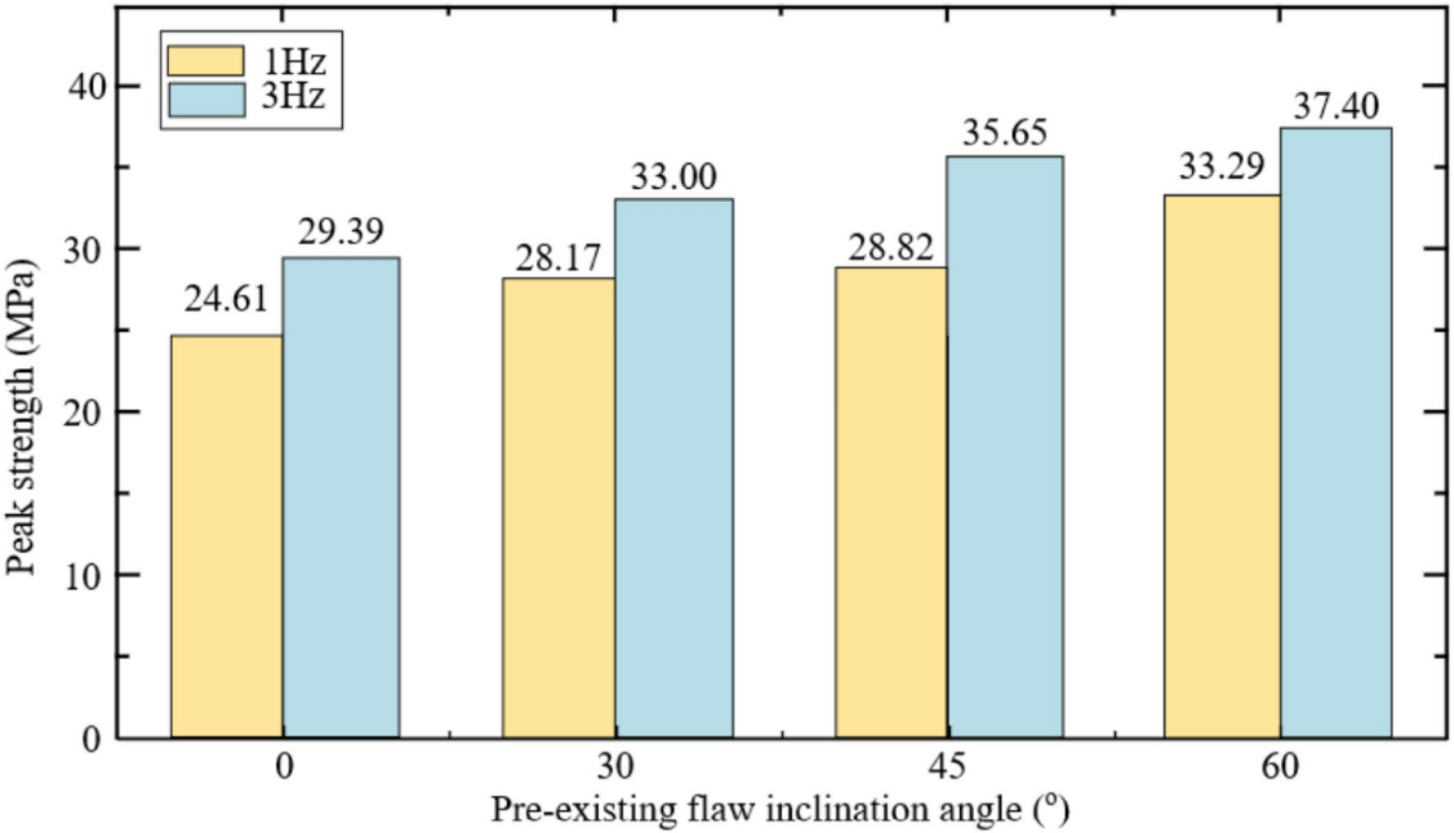
Effect of cyclic dynamic loading on peak strength.

Figure 19 shows that under cyclic dynamic loading, the compressive strength of the specimen increases to varying degrees. Notably, the specimens with *γ* = 60 are the most affected. The primary factors contributing to this phenomenon are as follows.

On the one hand, due to the applied axial load, stress concentration occurs at the tip of the prefabricated inclination angle of the specimen. For specimens with larger inclination angles, the rock column is larger and the bearing capacity is higher. Therefore, rock samples containing prefabricated joints with large inclination angles are less likely to fail under cyclic dynamic loading. On the other hand, as the load is continuously applied, the dynamic elastic modulus gradually increases, enhancing its ability to resist deformation and enabling it to better recover to its original state under dynamic conditions.

#### Crack propagation characteristics and fracture mode of specimens

**Figure 20:**
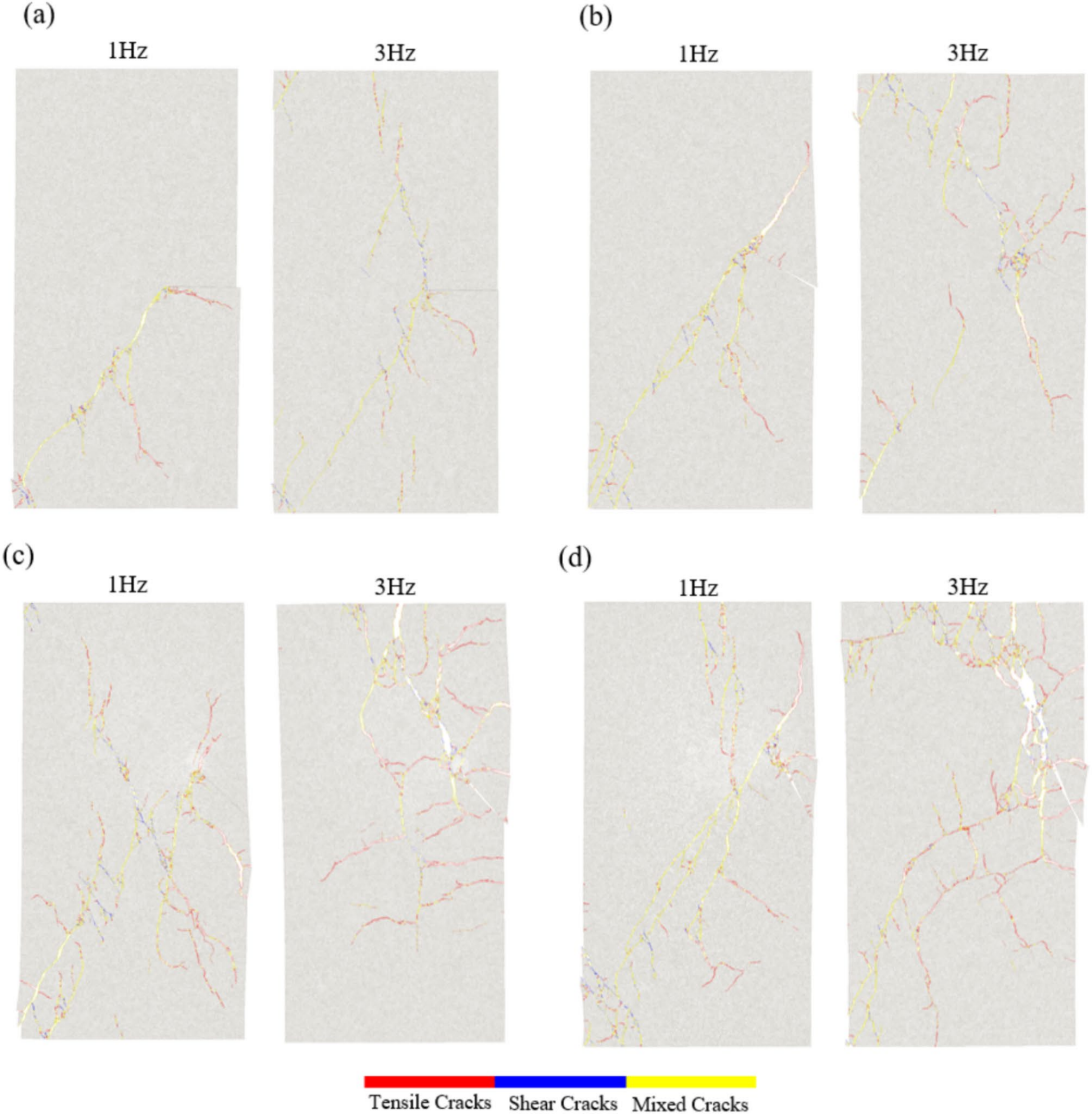
Failure modes of specimens with different inclination angles under cyclic dynamic loading. (**a**) *γ* is 0°, (**b**) *γ* is 30°, (**c**) *γ* is 45°, and (**d**) *γ* is 60°.

Figure 20 illustrates the varying crack propagation patterns due to the presence of pre-existing flows at different angles. When the rock samples have smaller inclination angles, the tips of the pre-existing flows tend to develop mixed-mode cracks. As the angle increases, the failure mode becomes more singular, making the specimens progressively more challenging to fail. High-frequency dynamic loading can induce faster sliding on both sides of the crack, accelerating the crack propagation rate and accompanied by particle debonding and local rock block loosening.

Figure 20 also shows that the failure modes of the rock under cyclic dynamic loading at different frequencies are distinct. Under a cyclic load of 1 Hz, the main fracture surfaces of the rock samples primarily exhibit mixed-mode crack distribution. In contrast, under a cyclic load of 3 Hz, the cracks on the main fracture surfaces are predominantly tensile cracks.

It can be observed that fatigue damage is difficult to observe when selecting the cyclic load applied in this article. The main reason is that the amplitude of the applied cyclic dynamic load is 0.5 mm. If the rock sample is to remain intact during the loading process, its strain should be 0.83%, which has exceeded its maximum strain.

Nevertheless, it is meaningful to study using a displacement time history with an amplitude of 0.5 mm as the input for dynamic loads. The main focus of this article is to study the dynamic characteristics of common fractured rock masses on the surface under cyclic dynamic loads, and displacement time history curve is one of the common seismic motion recording methods. The displacement amplitude of some strong earthquake records can often reach the level of cm.

## Conclusions

In this paper, a hybrid finite-discrete element method is proposed for modeling the cracking processes in sandy mudstone containing a single edge flaw under cyclic dynamic loading. The method's applicability is validated through comparative analysis with experimental results. Furthermore, based on this method, the mechanical properties and failure mechanisms of edge cut pre cracked specimens under quasi-static and cyclic dynamic loads were studied. The conclusions are as follows:

1. Under the action of quasi-static loading the stress–strain behavior of specimens with a single edge-flaw exhibits brittle failure. The compressive strength increases with an increase in the angle of the pre-existing flaw inclination. Mainly because as the angle increases, the effective bearing area also gradually increases.
2. When a cyclic dynamic load with an amplitude greater than the ultimate strain range that the specimen can withstand is applied to the rock sample, the compressive strength of the rock sample increases with the increase of inclination angle and dynamic load frequency.
3. Compared with the quasi-static loading, the cyclic dynamic loading intensifies the axial deformation of jointed rock mass. As the joint angle increases, the failure mode of the specimen under cyclic loading also changes, gradually transitioning from mixed failure to the tensile failure.

Therefore, the HFDEM model can serve as a powerful tool for investigating the development of rock cracks and the process of rock failure under seismic loading conditions.

## Data Availability

Data is provided within the manuscript or supplementary information files.
